# Why allergen detection by ELISA is insufficient to protect allergic consumers

**DOI:** 10.1186/2045-7022-3-S3-O9

**Published:** 2013-07-25

**Authors:** B Popping

**Affiliations:** 1 Scientific Development, Eurofins, Hamburg, Germany

## Background

In 2010, a multi-allergen detection method based on mass spectrometric (LC-MS/MS) analysis was developed as described in JCA1218. This method was able to demonstrate that seven allergens could be detected in a single analysis. Later, it could be shown that the same method is capable to detect allergens in processed materials while conventional methods like ELSIA and PCR failed in the same matrix, even when amounts as high as 1000mg/kg were present. This was reported in JAOAC 94/4. In mid-2012 a case of a finished product was reported, which caused allergic reactions in children. The product was analysed by ELISA and LC-MS/MS. The ELISA failed to detect the allergens while mass spectrometric analysis clearly demonstrated the presence. This is the first case where evidence suggests that conventional methods like ELISA and PCR are not sensitive enough in some processed materials to protect allergic individuals, while LC-MS/MS is still able to detect these allergens.

## Methods

To protect the allergic consumer, analytical methods need to be capable of detecting allergens in finished products that typically contain multiple allergens. An LC/MS/MS method for simultaneous detection of seven allergens was developed and compared with commercially available ELISA kits. The detection capabilities of this novel method were demonstrated by analyzing incurred material containing milk, egg, soy, peanut, hazelnut, walnut, and almond. Bread was chosen as a model matrix. To assess the influence of baking on the method’s performance, analysis was done before and after baking.

## Results

It was demonstrated that in the model matrix bread allergens in concentrations of 1000mg/kg for egg, soya and milk remained undetected by ELISA while the developed mass spectrometric method was well capable of detecting the allergens.

## Conclusion

A review of applicable methodology for the detection of allergens in different matrices is required to improve safety for affected consumers and to allow industry to make a meaningful risk assessment.

## Disclosure of interest

None declared.
